# Croatian 2008-2010 health insurance reform: hard choices toward financial sustainability and efficiency

**DOI:** 10.3325/cmj.2012.53.66

**Published:** 2012-02

**Authors:** Luka Vončina, Tihomir Strizrep, Mario Bagat, Dubravka Pezelj-Duliba, Nika Pavić, Ozren Polašek

**Affiliations:** 1 Croatian Institute for Health Insurance, Zagreb, Croatia; 2 School of Medicine, University of Zagreb, Zagreb, Croatia; 3 School of Medicine, University of Split, Split, Croatia; 4 Croatian Centre for Global Health, Split, Croatia

Over the past two decades, Croatian social health insurance system has undergone a number of reforms that were aimed at addressing the rising financial gap between the demand for health care and available public resources. The single most important observation regarding its development until 2008 is that, while attempting to provide quick fix solutions to worrying financial performance, the implemented reforms did not adequately take into account some of the root causes of the system’s sustainability issues, such as high public spending, a narrow base of financial contributors, generous benefits, and system inefficiencies that resulted in unnecessary expenditures and in low financial discipline. This text provides an overview of the segments of the reform of the Croatian social health insurance system that was implemented from 2008 to 2010 with two primary goals: financial stabilization and increasing system efficiency. These include changes introduced to the revenue collection mechanism, copayment levels, hospital and primary care payment models, introduction of a comprehensive primary health care information technology (IT) system, improved control of sick leave compensations, and advanced regulation of the pharmaceutical market. In order to properly review these measures, it is necessary to understand the context in which the social health insurance system existed until 2008.

## The Croatian Health care system until 2008

In the period from 2000 to 2008, as other Central European countries of comparable development and gross domestic product (GDP) per capita, Croatia spent a substantial proportion of its GDP on health care. However, in absolute terms, Croatia did not spend an excessive amount on health care per capita (1), with lower spending reported only in Hungary (2,3; Table 1). While available World Health Organization data show a decreasing share of public expenditure in the total expenditure on health between 2000 and 2008 in all selected countries, the share of public expenditure in Croatia has nevertheless been larger than in other countries, and comparable only to that of the Czech Republic (Supplementary Table 1>)(web extra material 1). The level of public spending on health relative to other countries is even better visible from public sector expenditure on health as percentage of total government expenditure (Supplementary Table 2)(web extra material 2). It may be argued that both indicators show the continued importance of health care on the Croatian policy-making agenda resulting in high public expenditure. Before Croatia declared independence in 1990, the former Yugoslav system had been organized at the regional level under the guiding principle of “self-managing socialism” that (albeit only nominally) provided universal and comprehensive coverage of all health services free at the point of use. Consequently, access to health care came to be viewed as a universal right of citizenship (1).

**Table 1:** Total health expenditure, expressed in US dollars per capita and the percentages of the gross domestic product (in the parentheses) (2,3)

Year									
	2000	2001	2002	2003	2004	2005	2006	2007	2008
**Croatia**	842 (7.8)	844 (7.3)	790 (6.3)	869 (6.5)	968 (6.7)	1071 (7.0)	1184 (7.1)	1398 (7.6)	1496 (7.8)
**Czech Republic**	980 (6.5)	1082 (6.7)	1195 (7.1)	1339 (7.4)	1422 (7.4)	1477 (7.2)	1535 (7.0)	1626 (6.8)	1684 (6.8)
**Hungary**	852 (7.0)	970 (7.2)	1114 (7.5)	1284 (8.3)	1305 (8.0)	1411 (8.3)	1452 (8.1)	1388 (7.4)	1419 (7.4)
**Slovakia**	720 (6.6)	837 (6.9)	922 (7.1)	1024 (7.5)	1057 (7.2)	1139 (7.0)	1322 (7.3)	1555 (7.7)	1717 (7.8)
**Slovenia**	1447 (8.3)	1581 (8.6)	1693 (8.6)	1772 (8.7)	1869 (8.4)	1986 (8.5)	2056 (8.3)	2099 (7.8)	2183 (7.8)

**Table 2:** Provider arrears in millions of HRK, 2000 to 2005 (16)

	Hospitals		All health care providers	
End of:	total debts	due arrears	total debts	due arrears
**2000**	1682	686	2142	758
**2001**	1893	877	2296	947
**2002**	1826	777	2307	865
**2003**	2269	1095	2778	1178
**2004**	2569	1108	3235	1406
**2005**	3213	1508	3887	1797

The Croatian health care system is financially dominated by the national social health insurance fund – the Croatian Institute for Health Insurance (Croatian: *Hrvatski zavod za zdravstveno osiguranje*, HZZO). The HZZO offers two separate health insurance packages. Mandatory health insurance (MHI) provides a basic generous package of services and products. Contributions are mandatory and are levied at 15% of gross salary (4). Access to services for the majority of the population is not entirely free at the point of use, but is subject to co-payments that prior to 2008 were modest, although enlarged on several occasions affecting the accessibility of the system, and a substantial part of the population was exempted. In addition, the HZZO offers its voluntary complementary health insurance (CHI) package with community-rated premiums that cover the expense of all co-payments. Private health insurance companies offer insurance packages that can cover co-payments and a higher standard of accommodation (hotel amenities in hospitals) in HZZO-contracted providers and products and services provided by private insurers (Supplementary Figure 1)(web extra material 11).

The bulk of public funds for MHI originate from one source: payroll contributions. In addition, a minor part of funds is collected by general taxation. Thus, as elsewhere in social health insurance countries, the funding of Croatia’s MHI system does not depend solely on salary contributions and it displays characteristics of both Bismarck and Beveridge systems (5). In effect, HZZO expenditure on health is prospectively determined for every subsequent year by forecasting the amount that will be collected from salary contributions, determining the amount of funds to be hypothecated for health from general taxation, and negotiations on expenditure between the Ministry of Health and Social Welfare, the HZZO, and the Ministry of Finance.

Contributions for MHI (at a uniform rate of 15% of gross income) have since 2002 been levied on both salaries and honoraria. Subsidization of the non-contributing insured through funds from general taxation was until 2008 loosely regulated by the Health Insurance Act, which determined that it was an obligation of the state to ensure funds for medical treatment and cash compensations for certain categories of the population that do not financially contribute to MHI (such as the unemployed registered with the state Employment Office, students and high-school pupils, drafted soldiers, uninsured Croatian war veterans and their caregivers, farmers over the age of 65, disabled, etc.), but did not specify it in more detail (6).

Croatian social health insurance system faces several long term trends that threaten the economic sustainability of revenue collection: a low percentage of those who actively financially contribute to the MHI fund (the employed) compared to the total number of beneficiaries, the aging of the Croatian population caused by long standing decreases in natality, fertility, and natural increment, with almost 15.7% of the population aged 65 and over in 2001 (7); and in the last five decades the consequent increase in the percentage of persons aged 65+ who do not financially contribute to the system, and are its heaviest spenders. Furthermore, the proportion of the total population that actively contributes to the MHI is around 36% (8,9; Supplementary Table 3)(web extra material 3).

Historically, until 2008 the government made little effort to diversify the revenue base for the health sector. Revenue generation of the MHI was generally limited by payroll tax as its dominant collection mechanism by which only one third of members made contributions. Government budget transfers from general revenues to the MHI fund have been variable until 2008 and heavily dependent on the economic situation. Thus, the fund-raising system for HZZO’s MHI was made increasingly dependent on salary contributions, and consequently on the employment ratio and wage level – which until the recession enjoyed favorable trends. Contributions for MHI accounted for more than 95% of mandatory health insurance expenditure in 2007, compared with 84% in 2002 (10-14; Supplementary Table 4)(web extra material 4).

Fundamental challenges for the Croatian government during times of economic crisis have been similar to those of other European countries: how to expand the health care system’s funding base and prevent health care costs from outgrowing the funding base. In the last couple of decades, overall health care expenditures of the leading European countries (Switzerland, France, Italy, Germany, Spain, and the United Kingdom) have grown on average by about 80%, while the GDP of the group has grown only by 25%. Since the beginning of the global crisis, health care expenditures in the above mentioned countries continued to outpace GDP and wages. This problem was surely aggravated by unemployment generated during the recent economic recession, which adversely affected the dependency ratio – that is, the ratio between the number of people who contribute economically to the social welfare system and the number who receive benefits. And the dependency ratio is expected to deteriorate further in most European countries over the next decade (15).

In order to receive public funds for providing health services in Croatia, all providers regardless of ownership are required to enter into annual contracts with the HZZO that set prices for services and mechanisms of payment. In the past, the HZZO tried to limit its expenditure on hospital-provided care through hard budgets, gatekeeping, and limitations on the number of allowed referrals for general practitioners (GP) and other doctors employed in primary health care; and its expenditure for primary care through the capitation payment system. Regardless, due to the social sensitivity of hospital health care, annual hospital hard budgets have in the past often been enlarged during on-going years, ie, on four occasions in 2003 (10). Besides having a direct influence on HZZO’s expenditure, these created detrimental incentives toward HZZO and, particularly, hospital efficiency as they created a widespread impression that the government would cover all deficits, without considering how these were created. For illustration, from 2000 to 2005, despite several state interventions that poured over HRK 4.2 billion of funds into the system for the settlement of arrears in health care providers, the amount of total debts of health care-providing institutions grew by staggering 81.5%, while the amount of due arrears grew by even higher 137% (Table 2). The majority of obligations (62% in 2005) accounted for drugs and consumables (16), threatening their undisrupted delivery to the population.

### Primary care

Monitoring and regulation of GPs’ referrals and pharmaceutical expenditure lacked sophistication and were difficult to implement in practice – in major part due to the absence of IT solutions that would facilitate control. Furthermore, the privatization of primary health care and payment by capitation – introduced in 1993 as a simple solution to control primary care costs – failed to financially stimulate GPs to be efficient. Primary care physicians were paid on the basis of flat fees per patient per year regardless of the volume and quality of services provided. The system created incentives for GPs to accumulate as many patients as possible, prohibiting them from providing efficient quality care (17). Primary care capitation, “matched” with fee-for-service payments for hospital specialist examinations and inpatient care, meant that both primary care physicians and upper-end providers had financial incentives to see patients referred up the delivery system, instead of managing more patients at the primary care level. As a result, the proportion of inpatient spending and hospital admissions had to increase (18).

Nevertheless, a study analyzing the preliminary effects of the privatization process for primary health care in Croatia did find some positive effects. Privatized practices performed better in improving access to their services for patients: they increasingly offered the possibility for first and follow-up appointments at precise times, scheduled visits by telephone, and provided telephone advice outside working hours. They also showed greater intention to honor appointment times in order to lower their patients’ waiting times (19).

In terms of cost containment efforts and their role as gatekeepers, primary care physicians play an influential role in determining the overall costs of health care (20). In line with what was discussed above, reports from the Croatian National Institute of Public Health indicate substantial reductions in numbers of rendered preventive services and home visits as well as large increases in numbers of (necessary or unnecessary) referrals to secondary and tertiary health care.

In 2008, the recorded number of home visits was 290 075, a decrease of 47% compared to 1990. Additionally, there were 7.491 million referrals from primary care physicians, meaning that the number of referrals for secondary and tertiary care grew by 49% from 1995. Data for primary care service for 2008 show that for every 2.1 regular checkups for adults there was one specialist referral. These indicators show that primary health care turned into a passive health care service that failed to deliver significant impact on the positive health outcomes for the population and that the capitation fee system was not efficient (21).

### Secondary and tertiary care

Hospital services in Croatia were funded through monthly allocations of the annual hospital hard budget, controlled by the HZZO as the paid funds had to be accounted for by bills for medical services in a combination of a point-based hospital payment system and a diagnostic-related groups (DRG) system referred to as the payment per therapeutically procedure (PPTP). If a hospital exceeded its annual budget ceiling, it did not receive funds for the bills for services provided once the ceiling was reached. Additionally, hospitals were allowed to charge full payments to those without insurance, and co-payments for the MHI-insured which were neither exempt nor had complementary insurance.

The point-based hospital payment system was essentially a fee-for-service (FFS) reimbursement system in which hospitals were reimbursed on the basis of inputs rather than outputs or outcomes. The hospital payment system consisted of three separate components: 1) hotel services, paid by a flat *per diem* payment, 2) services, and 3) pharmaceuticals and other materials that were paid separately, depending on the cost of each item. Under the FFS system, hospitals had an incentive to maintain a high level of bed occupancy and extend length of stay, since this high occupancy resulted in stable funding through *per diem* payments, while the majority of costs tend to be concentrated in the first few days of hospital stays. Low occupancy rates also increased the risk that the HZZO would lower the global budget ceiling (22).

In 2002, the government started introducing a parallel DRG-based payment system (Croatian: *plaćanje po terapijskom postupku,* PPTP) that used broad case groupings. Interventions for these cases were either costly or numerous and the prospective payment system was intended to provide hospitals with incentives to increase the technical efficiency of service provision (23). In effect, the HZZO used hospital budgets as an expenditure and volume of services control mechanism. However, the negotiation of hospital budgets ceilings appeared to be a sometimes controversial and insufficiently transparent issue as some hospitals struggled to charge enough services to reach their limit, while others struggled to stay under the limit. For example, in 2005 a part of Croatian hospitals exceeded their budgets by HRK 111 million, while the other part charged HRK 121 million less than their budgets allowed them to (24). It appears that while, in general, smaller county hospitals had problems in supplying enough services to reach their budget limits, bigger hospitals located in larger cities had problems in staying within budgets. This may have been due to the government’s dedication on maintaining the *status quo*, or in other words, keeping the smaller hospitals in business rather than reducing them in size, or even closing some down. While this policy may have been fully legitimate on a national level, a more transparent way of subsidizing some of the smaller hospitals may have been worth looking into. The issue was further blurred by the fact that it was hard to determine whether hospitals were overcharging or undercharging for care as under the FFS schedule they were able to charge substantially different amounts for treating similar patients. In effect, the payment model was awarding inefficiencies. Comparisons of performance indicators with other central European countries clearly demonstrate this policy failure (25; Supplementary Table 5)(web extra material 5).

The use of broad-based case groupings in the PPTP system, as opposed to more detailed DRGs, and the prices set for particular PPTP groupings have made the system quite unpopular with providers. Charging services according to the FFS system seemed to be far more lucrative than according to the PPTP system, which has often been accused of underestimating actual costs. Although, legally, these should have not be interchangeable, reports on billing practices indicate that hospitals may have been engaged in a large scale creative accounting practice (ie, cheating) as they charged only 30% of services according to the sometimes less lucrative PPTP schedule, while epidemiologic arguments suggested that PPTS schedule should have covered over 60% of treated conditions and diseases (26). Finally, PPTP provided incentives for cream-skimming. Hospitals were able to attempt to avoid high-risk, high-cost patients by “dumping” them on other providers. An analysis of the Croatian Institute of Public Health showed that patients rarely use hospitals in their counties (25). It should also be noted that general hospitals (county hospitals) were allowed to refer cases that were judged to be clinically complicated to clinics/clinical hospital centers that provided state-of-the-art treatment, and received equal payment as they would for less complicated cases.

### Pharmaceutical market

Similarly to most European countries, over the recent decades Croatia has witnessed fast increase in the demand for medicines. Such situation warranted the use of foreign experiences, which were often of limited usefulness as governments have often implemented different measures in rapid succession or even simultaneously (27,28). Key discussions in Croatia mostly related to limiting the range of pharmaceuticals eligible for payment or reimbursement under the mandatory and complementary insurance schemes; and to proposals that the insured should be called to carry a larger part of the financial burden of pharmaceutical expenditure.

In 2004, the Croatian government introduced a new system of setting maximum prices for drugs, which resulted in price cuts of up to 15% on many imported pharmaceuticals. The system took into account drug prices in Italy, France, and Slovenia (for example branded drugs had to be 10% cheaper than the price average of the 3 mentioned countries). The choice of the latter country was significant, as Slovenia at that time had a mechanism of setting local wholesale prices at 85% of the price of new pharmaceuticals in France, Germany, and Italy. Prices could have been, however, also determined by direct negotiation with suppliers. In 2006, the government introduced internal reference pricing. The government set limits to the reimbursement level for all drugs by using the existence of lower priced generic drugs on the market. The reference price for all generically equivalent drugs was fixed at an amount that the authorities regarded as acceptable. If the price of any product was higher than the reference price, MHI payment or reimbursement would only be granted up to the level of the latter, and the difference between this and the actual market price would have to be paid by the patient (“co-payment principle”). However, until 2007 HZZO’s CHI scheme covered the cost of this co-payment, limiting the incentives that internal reference pricing brought to pharmaceutical companies toward decreasing prices to remain free at the point of use. This policy on the other hand contributed to CHI’s poor financial results, for instance it ended 2006 with a deficit of HRK 32.76 million (13). As of 2007, CHI no longer covered the medicines’ co-payment and ended the financial year with a surplus of HRK 665 million (14).

Until 2008, expenditure on prescription medicines grew at a fast pace, amounting to HRK 3.098 billion in 2004, 3.116 billion in 2005, 3.248 billion in 2006, 3.144 billion in 2007, and 3.391 billion in 2008 (8,11-14). Expenditure on prescription drugs decreased in 2007 compared to 2006, which implies that the reform had a positive impact on overall costs. However, the decrease was obviously temporary since the expenditures rose once again in 2008. This leads to a conclusion that the 2004 and 2006 health care reforms failed in preserving long term budget balance by containing overall costs.

### Balance of payments

Even the simplest analysis of the balance of payments in the 2002-2008 period indicates that in nearly all years HZZO was producing deficits (8-14), suggesting that reforms and measures implemented in the period failed to prove effective in the long term (Table 3). An analysis of the dynamics of deficits and arrears in the Croatian health care system up to 2008 points to several issues. The first and most obvious one is that almost every year more funds were spent than the state hypothecated for health care, which points to a mismatch between the desired level and mix of objectives and the limited capacity to pay. The second issue is that annually both the HZZO and the providers overspent their budgets, which indicates that their budgets could better be described as soft budgets, rather than hard budgets – which they were intended to be, a conclusion that implies low fiscal discipline in the system. To preserve financial liquidity, both delayed payments to suppliers and so created arrears. Allowing this practice has created a vicious circle in which arrears skyrocketed and delays grew. In 2008, total accumulated arrears reached HRK 4.8 billion. Dynamics of generating new debts was HRK 200 million a month. In 2008, the HZZO reimbursed pharmaceutical wholesalers with over 290 days of delay (29). The financial issues were additionally aggravated by the sick leave rate with its expenditure in 2007, amounting to HRK 1.35 billion (14).

**Table 3:** Croatian Institute for Health Insurance’s deficits (surplus) from 2002 to 2008, in million HRK

	Revenues	Expenses	Difference
**2002**	14 181	13 857	+324
**2003**	14 590	14 718	-128
**2004**	14 687	15,857	-1170
**2005**	15 565	16 157	-592
**2006**	16 897	17 389	-492
**2007**	19 143	18 411	+733
**2008**	20 644	20 736	-92

## The 2008 reform

The 2008 reform focused on financial stabilization and increasing system efficiency. Measures included changes introduced to the revenue collection mechanism, copayment levels, hospital and primary care payment models, introduction of a comprehensive primary health care IT system, improved control of sick leave compensations and advanced regulation of the pharmaceutical market. The necessity of the measures was primarily argued as the government at that point had to secure funds additional to those already hypothecated for health care in 2008 to ensure undisrupted delivery of health care services. These amounted to HRK 459 million for hospital arrears settlement, HRK 800 million for prescription drugs arrears settlement, and HRK 100 million for expensive drugs debt settlement (29).

### Financial stabilization

Main features of the financial component of the reform focused on diversification of public revenue collection mechanisms, aligning the public private split in funding to those of central European countries through increasing copayment levels and reducing the extent of categories exempted from copayments, introducing stringent financial discipline, and resolution of accumulated arrears (HZZO and hospitals; Table 4).

**Table 4:** The 2008 financial reform measures

Measure	Specific actions
Diversification of public revenue collection mechanisms through introduction of new mandatory health insurance (MHI) and complementary health insurance (CHI) contributions	MHI contributions for the retired with pension under average salary paid from general taxation: rate 1% of gross pension Retired with pension over average salary pay MHI contributions: rate 3% of gross pension MHI contributions for the unemployed paid from general taxation: rate 5% of fixed sum MHI contributions for pupils, students, war veterans, soldiers, asylum seekers, etc. paid in bulk from the state budget Hypothecated cigarette taxation: 32% of excise duty Tax on mandatory car insurance premiums: 7% (to cover the cost of health care provided due to traffic accidents) CHI contributions for 100% disabled, organ donors, multiple blood donors, pupils, students, and all individuals with income per household member under the national poverty census threshold – paid from general taxation
Increased copayments	Inpatient and outpatient hospital services: 20% of price (previously 15%-50%) Dentistry: 20% of price (previously 15%-50%) Primary care family medicine and gynecology: HRK 15 per visit deductible Prescriptions – HRK 15 per prescription deductible Price cap for all copayments: HRK 3000 per episode of illness
Reducing population exempted from copayments	360 000 citizens became eligible for copayments (unemployed, war veterans, disabled with disability under 100%, etc.) Exempted populations: children, pregnant women, patients with HIV, chronic psychiatry patients, transplant patients, dialysis and cancer patients, citizens living under the poverty level
Complementary health insurance	Price increase from HRK 50 to 80 per month for the best of retired and HRK 80 to 130 for employed with large salaries
Financial discipline	Stringent control of expenditure on all levels of the system
Resolution of accumulated arrears	Rationalization of expenditure Monitoring of debts and arrears Ban on increasing arrears

The financial effect of the reform on public resources can be seen from the expenditure allocations recorded in the national state budget. Compared to 2009, in 2011 the Ministry of Health and Social Welfare reduced its expenditure by HRK 304 million (7.3%), and the HZZO (including HZZOZZR) by HRK 2.15 billion (9%) cumulating total savings of HRK 2.46 billion (29; Supplementary Table 6)(web extra material 6). An additional financial effect of the reform measures is revealed through the reduction of HZZO and hospital arrears (29; Supplementary Table 7)(web extra material 7) – a reduction of 60%, from HRK 4.8 to 1.9 billion. Thus, despite the reductions in state budget allocations, the system was able to further constrain expenditure so as to liquidate the majority of its arrears. The financial savings of the reform from the perspective of public finances are primarily due to a) tackling system inefficiencies (rationalization of expenditure) and b) increasing the private proportion of funding. While the former will be elaborated in more detail later in the text, between 2008 and 2010 the total amount of copayments paid in primary and secondary care increased 2-fold, from HRK 651 million to 1.4 billion (29).

The issue of social protection has been addressed through a copayment ceiling for episode of illness (HRK 3000), affordable community-rated CHI premiums (ranging from HRK 600 to 1560 per year depending on income), and exclusion of certain vulnerable categories of the population from copayments. As could have been expected, the rise of copayments and the widening of the population categories eligible for copayments made CHI substantially more popular and its number of insured rose from HRK 710 359 in 2008 to 2.7 million in 2010 (8,30). Of these, 1.4 million paid premiums out of pocket, the remaining other 1.3 million were insured by premiums paid from the state budget (30).

Apart from the greater reliance on private spending, diversification of revenue sources has been achieved through more explicit determination of the state’s obligations on behalf of the non-contributing insured: MHI contributions for the retired and the unemployed, CHI contributions for the mentioned categories, cigarette taxation, and car insurance tax. While these obligations were in the past paid arbitrarily, usually through additional funds for debt settlement, their more explicit determination guaranteed that the health sector would be able rely on them in the years to come, regardless of short-term government priorities and financial situation, and that these would relieve the pressure of the labor market and the health care system’s dominant revenue source – payroll contributions. Bulk sum transfers from the state budget on behalf of MHI contributions for pupils, students, war veterans, soldiers, asylum seekers, etc, amounted to HRK 208 million in 2009. These have not been settled in 2010 due to the economic recession and as the total sum of public revenues other than MHI contributions grew from HRK 2 to 2.4 billion compared to 2009 (Supplementary Table 8)(web extra material 8), which further testifies of the importance of the above mentioned. The implemented measures enabled the government to financially consolidate the system without limiting the benefits package, ie, the scope and quality of health care services provided.

### Primary health care IT system: e-primary health care

As of January 2011, e-prescriptions and e-referrals to biochemical laboratories in family medicine became fully functional in all Croatian counties. IT in health care has the potential of bringing about substantial savings related to efficiency and improvements in quality (31). Croatian e-primary health care project enables secure, real-time exchange of data between all primary health care stakeholders and hospitals. The system was primarily designed to improve and simplify the delivery of health care to patients – patients no longer have to collect their laboratory findings as these are delivered directly to doctors, doctors are informed of dispensing of prescribed medicines so that they can monitor compliance, etc. Main benefits for health professionals include substantial relief from administrative tasks, which are now fully automated, and improved communication with other stakeholders in the system. Health care authorities benefit from significant savings from printing prescriptions and referrals, productivity and efficiency gains, automated checking of all insurance data, and real-time information that enables informed decision-making with the aim of increasing the efficiency and equity of health care provision, such as for example prescribing or referral patterns, etc.

### Primary health care reform

The health care reform introduced several structural and financial changes to primary health care. The system was decentralized through the introduction of concessions – tenders that county governments organize for primary care specialties in the frame of the national network for teams operating outside of primary health care centers in which doctors work as salaried professionals. These have empowered counties (local authorities) to play a more active role in the organization, coordination, and management of their respective primary health care systems with the aim of better tailoring these to local needs. Geographic equity of access has been ensured by standardizing the number of capitated patients per team. Furthermore, the maximum and minimum number of patients allowed per team was determined at the level ±25% from the standard number with a three-year transitional period for compliance (33; Supplementary Tables 9 and 10)(web extra material 9).

Alongside the implementation of concessions, the reform entailed a change in the primary health care provider payment mechanism, introducing a fee for service payments and payments for special programs (preventive medicine and 24/7 open family medicine centers). GPs now earn 80% of income through capitation, while the remaining 20% of income depends on their activities. While data on activities (preventive checkups, referrals, etc.) are as of yet not available, the new financial incentives should ensure improved primary care productivity and efficiency in the years to come. Finally, a new incentive payment system was introduced for primary health care centers to fill vacancies on islands, and in rural and less developed areas to improve geographical equity of access.

HZZO’s total expenditure on sick leave compensations in 2010 amounted to HRK 1.1 billion, compared to HRK 1.22 billion in 2009, which led to savings of over 108 million HRK. The savings are due to stricter controls of primary care physicians prescribing sick leaves introduced in 2009. In 2010, the total number of sick leave days amounted to 14.765 million working days, 1944 million fewer than in 2009, considerably reducing costs, but also contributing to the overall productivity of the Croatian economy (30).

### Hospital payment – diagnosis related groups

Encouraged by reports of efficiency gains arising from the implementation of the PPTP schedule, including reductions in length of stay, the government decided to gradually move toward a comprehensive prospective case-adjusted payment system based on DRGs. As in some other European countries, such as Ireland, Romania, Germany, and Slovenia, Croatia has decided not to develop its own DRG system, but rather to import and modify the Australian Refined-DRG (AR-DRG) system (specifically, version 5.1), in Croatia known under the abbreviation DTS (*Croatian: Dijagnostičko terapijske skupine*) (23). The DTS payment system was fully implemented on January 1, 2009. Payment results of the DTS payment system (length of stay, costs, etc, by DRG) have since then been published monthly for all hospitals on HZZO’s Web site, enabling benchmarking.

The main intention of the DTS payment system was to enable cost reduction and rationalization, as well as affect concrete performance indicators such as shorter hospitalization time (average length of stay per hospitalization), and thus higher patient turnover, which would reduce waiting lists for certain procedures. Despite the slight increase in length of stay from 2009 to 2010, which can tentatively be explained by its sharp drop in the first year of implementation, the DTS system has proved successful in decreasing length of stay in both university and general hospitals (Supplementary Table 11)(web extra material 10).

### Pharmaceutical pricing and reimbursement reform

In 2009, Croatia substantially reformed its pricing and reimbursement regulation for medicines with the aim of maximizing value for invested funds, increasing the efficiency and transparency of high level decision-making, and ensuring ethical pharmaceutical marketing practice (32). Most notably, the reform measures include defined judgment criteria and full public disclosure of the reimbursement decision-making process, pricing reforms, strengthening of evidence-based medicine and health economic requirements for submissions, pay back, rebate, and cross product agreements, mandatory reporting of promotional expenses, and all financial transactions between pharmaceutical companies and doctors employed by the public health care system, etc. The results of the reform enabled HZZO to generate extensive savings while at the same time improving access to innovative medicines (33).

Total HZZO’s expenditure on prescription medicines in 2009 amounted to HRK 2.9 billion, with additional 2 billion spent on hospital medicines (of which 480 million were spent on expensive products that are funded from a separate budget above regular hospital budgets). Due to the introduction of modest copayments (HRK 15 per prescription) and reference pricing, HZZO’s expenditure on prescription medicines decreased by 2.9% in comparison to 2008 (34). HZZO’s due arrears for medicines also substantially decreased in the time period, from HRK 1.3 billion to 1 billion, with a reduction of 22% (35).

Additionally, all applications to the HZZO’s lists became public. All applications are published on HZZO’s web page within five work days following the day of their receipt. Membership of the HZZO’s Committee for Medicinal Products and the timing and agendas of the regular sessions also become public. The methodology in which the committee reaches its recommendations was advanced and made more transparent. The committee now operates in two semi-annual cycles. The cycles consist of four regular sessions where the Committee discusses the submitted applications and of the fifth regular session where it ranks the applications that may increase HZZO’s expenditure on drugs. Ranking is undertaken using Delphi – a consensus building method making sure that all members of the committee carry equal weight in the decision making process. In addition, the new ordinances introduced detailed criteria based on which the committee is to reach its recommendations. These include the following: 1) importance of a medicinal product from the public health viewpoint; 2) therapeutic importance of a medicinal product; 3) relative therapeutic value of a medicinal product; 4) assessment of ethical aspects and 5) quality and reliability of data and assessments from reference sources.

The requirements for the applications based on which the committee makes its recommendations have also been raised. Most importantly, these (among others) include (36) 1) a tabular presentation of the status of the product in health insurance or health care system of all Member States of the European Union and, if available, a decision or opinion about financing of the product issued by the competent authority engaged in the assessment of health care technology, along with indications and instructions for use, amount covered by the compulsory health insurance of each State, amount of surcharges and other information relevant for financing of the medicinal product in individual Member States; 2) scientific evidence demonstrating the advantages of the medicinal product for suggested indication(s) over the comparators, and primarily over medicinal products already included in the basic or in the supplementary reimbursement list of the Institute (meta analyses and systematic appraisals where available); and 3) therapeutic guidelines of Croatian and European expert associations for indications for which an application has been submitted. All applications for reimbursement of original products have to be accompanied by budget impact analyses. These are undertaken according to strict criteria that largely adhere to International Society for Pharmacoeconomics and Outcomes Research’s “Principles of Good practice for Budget Impact Analysis.

Internal reference pricing became better regulated. Groups are formed at the third or higher anatomical therapeutic chemical level. Reference prices are determined (in major part by taking account of price by defined daily dose) by unit dosage form for same or similar pharmaceutical forms, for each strength of the active substance and each pack size separately. Reference prices are determined on the basis of the lowest price of a product which recorded at least 5% of sales within a therapeutic group over a 12-month period preceding the reference pricing process. This principle was adopted to avoid the possibility of market shortages.

All applicants to the lists are also obliged to enter into a uniform Agreement on Ethical Promotion of Medicines with substantial financial penalties for unethical promotion. Main features of the agreement include 1) mandatory reporting of all promotional expenses and financial transactions between companies and doctors employed by the public health care system; 2) ban on advertising and distribution of prescription drugs to the general population; 3) ban on informing the general population on ongoing applications to avoid unethical pressure on the HZZO’s medicines committee; 4) ban on promotion targeted at doctors based on information that has not been scientifically proven; 5) ban on financial remuneration and remuneration of any kind to doctors for prescribing; 6) representation costs have been limited to HRK 1000 per doctor (does not include education); 7) individual sales representatives are allowed 15 minutes contact time per doctor per month.

## Conclusion

In conclusion, while the previous reforms of the Croatian health care system paid little attention to root causes behind the system’s financial unsustainability issue, the 2008 reform tried to address these through a set of coordinated measures targeted at both the demand and supply side of the system. Its importance is highlighted by the economic recession Croatia has undergone in the recent past that has made it hard to pursue alternative directions as it has seriously affected the government’s ability to generate additional funds for the system. The implementation of the reform required tough choices such as substantially increasing private funding to the level of other Central European countries, but also coherent and sophisticated measures targeted at resolving system inefficiencies such as improving pricing and reimbursement regulation for medicines, changes to the primary care capitation model, the introduction of DRGs in hospitals and information technology in primary health care, etc. All represented changes to the inherited *status quo* and as such generated stiff opposition from system stakeholders. Nevertheless, governments as long-term stewards have the responsibility of taking tough decisions. The Croatian 2008 reform did this, and future health reforms should build on its achievements to further improve the regulation of the system. Finally, its success has been recognized by international institutions such as the World Bank having in mind the improvements it introduced in fiscal sustainability, reductions in outstanding arrears, as well as investments in improved patient access (37).
