# Supplementary material for: Croatian 2008-2010 health insurance reform: hard choices toward financial sustainability and efficiency
Source: Croat Med J. 2012 Feb;53(1):66–76. doi: 10.3325/cmj.2012.53.66 (PMC3284176; doi:10.3325/cmj.2012.53.66)
Supplement: Supplementary Table 1 [file CroatMedJ_53_s001.pdf]

Supplementary Table 1. Public sector health expenditure as % of total health expenditure, WHO estimates. Source of information: reference (2)

Year	2000	2001	2002	2003	2004	2005	2006	2007	2008
**Croatia**	86.1	83.3	80.0	82.6	81.1	86.0	86.1	87.0	84.9
**Czech Republic**	90.3	89.8	90.5	89.8	88.2	87.3	86.7	85.2	84.7
**Hungary**	70.7	69.0	70.2	72.8	72.4	72.3	72.5	70.6	70.3
**Slovakia**	85.0	84.7	86.3	74.8	73.8	74.4	68.3	66.8	66.8
**Slovenia**	74.0	73.5	73.4	72.0	73.0	72.0	72.5	71.5	71.4
