# Supplementary material for: Croatian 2008-2010 health insurance reform: hard choices toward financial sustainability and efficiency
Source: Croat Med J. 2012 Feb;53(1):66–76. doi: 10.3325/cmj.2012.53.66 (PMC3284176; doi:10.3325/cmj.2012.53.66)
Supplement: Supplementary Table 2 [file CroatMedJ_53_s002.pdf]

Supplementary Table 2. Public sector expenditure on health as % of total government expenditure, WHO estimates. Source of information: reference (2)

**Years**	**2000**	**2001**	**2002**	**2003**	**2004**	**2005**	**2006**	**2007**	**2008**
**Croatia**	14.5	13.8	12.0	14.0	14.1	15.9	16.4	17.6	17.6
**Czech Republic**	14.1	13.5	13.8	14.1	14.4	14.1	13.8	13.5	13.5
**Hungary**	10.6	10.4	10.4	12.3	11.9	12.0	11.3	10.5	10.5
**Slovakia**	10.7	13.2	13.6	14.0	14.1	13.8	13.6	15.1	15.0
**Slovenia**	13.1	13.3	13.6	13.4	13.4	13.5	13.5	13.2	13.2
