# Supplementary material for: Croatian 2008-2010 health insurance reform: hard choices toward financial sustainability and efficiency
Source: Croat Med J. 2012 Feb;53(1):66–76. doi: 10.3325/cmj.2012.53.66 (PMC3284176; doi:10.3325/cmj.2012.53.66)
Supplement: Supplementary Table 1 [file CroatMedJ_53_s003.pdf]

Supplementary table 3. Insured by categories in 2008. Source of information: reference (8)

	**Total**	**Proportion**
**Active persons in employment**	1,582,261	36%
**Active farmers**	52,685	1%
**Pensioners**	1,034,635	24%
**Unemployed**	376,447	9%
**Other insured persons**	126,560	3%
**Family members**	1,190,489	27%
**Refugees**	2,148	0%
**Total**	4,365,225	100%
