# Supplementary material for: Croatian 2008-2010 health insurance reform: hard choices toward financial sustainability and efficiency
Source: Croat Med J. 2012 Feb;53(1):66–76. doi: 10.3325/cmj.2012.53.66 (PMC3284176; doi:10.3325/cmj.2012.53.66)
Supplement: Supplementary Table 4 [file CroatMedJ_53_s004.pdf]

Supplementary Table 4. HZZO's MHI income and expenditure in million HRK. Source of information: references (10-14)

Year	**2002**	**2003**	**2004**	**2005**	**2006**	**2007**
**HZZO expenditure on MHI**	13,710	14,220	15,259	15,583	16,827	17,957
**HZZO income from MHI contributions**	11,533	12,324	13,271	14,165	15,400	17,056
**Coverage of HZZO's MHI expenditure by MHI contributions**	84,1%	86,7%	87.0%	90,1%	91,5%	95.0%
