# Supplementary material for: Croatian 2008-2010 health insurance reform: hard choices toward financial sustainability and efficiency
Source: Croat Med J. 2012 Feb;53(1):66–76. doi: 10.3325/cmj.2012.53.66 (PMC3284176; doi:10.3325/cmj.2012.53.66)
Supplement: Supplementary Table 5 [file CroatMedJ_53_s005.pdf]

Supplementary Table 5. Acute inpatient care, average length of stay and bed occupancy rates, 2008. Source of information: reference (25)

**Country**	**Average length of stay per hospitalization in days, acute inpatient care**	**Bed occupancy rates, acute inpatient care**
**Croatia**	7.36	83.08
**Czech Republic**	7.40	69.70
**Slovakia**	6.90	67.50
**Hungary**	5.95	75.29
**Austria**	5.80	80.40
**Slovenia**	5.68	71.50
**EU (2007)**	6.74	76.98
