# Supplementary material for: Croatian 2008-2010 health insurance reform: hard choices toward financial sustainability and efficiency
Source: Croat Med J. 2012 Feb;53(1):66–76. doi: 10.3325/cmj.2012.53.66 (PMC3284176; doi:10.3325/cmj.2012.53.66)
Supplement: Supplementary Table 6 [file CroatMedJ_53_s006.pdf]

Supplementary Table 6: State budget allocations for health care, in billion HRK. Source of information: reference (29)

State budget	Ministry of Health and Social Welfare	HZZO	HZZOZZR	Total
2009	4.172	23.507	0.394	28.073
2010	3.934	22.327	0.346	26.607
2011	3.868	21.741	0	25.609

Note: in 2011 the HZZOZZR (Croatian Institute for Health Insurance of Occupational Health) in charge of health insurance related to work accidents and professional illnesses was merged with the HZZO
