# Supplementary material for: Croatian 2008-2010 health insurance reform: hard choices toward financial sustainability and efficiency
Source: Croat Med J. 2012 Feb;53(1):66–76. doi: 10.3325/cmj.2012.53.66 (PMC3284176; doi:10.3325/cmj.2012.53.66)
Supplement: Supplementary Table 7 [file CroatMedJ_53_s007.pdf]

Supplementary Table 7: Reduction of HZZO and provider arrears, in billion HRK. Source of information: reference (29)

Year	HZZO arrears	Hospital arrears	Other providers' arrears	Total arrears
2008	2.064	2.268	0.467	4.798
2011	0.174	1.377	0.347	1.898
