# Supplementary material for: Croatian 2008-2010 health insurance reform: hard choices toward financial sustainability and efficiency
Source: Croat Med J. 2012 Feb;53(1):66–76. doi: 10.3325/cmj.2012.53.66 (PMC3284176; doi:10.3325/cmj.2012.53.66)
Supplement: Supplementary Table 8 [file CroatMedJ_53_s008.pdf]

Supplementary Table 8: HZZO's revenues additional to MHI contributions

	2008	2009	2010
HZZO – own revenue			
MHI contributions retired (3%)	0	48,214,910	45,977,245
Non Croatian citizens	216,679,288	264,559,974	404,239,150
Car insurance	0	125,791,417	174,852,530
Primary care copayment	101,091,259	533,431,836	493,886,222
CHI premiums	501,159,596	1,091,487,075	1,056,626,494
Total	818,930,143	2,063,485,212	2,175,581,641
Revenue from general taxation			
MHI contributions unemployed	0	103,809,000	484,909,497
MHI contributions pupils, students, war veterans, soldiers, asylum seekers, etc.,	0	208,239,423	0
MHI contributions retired	0	264,246,000	367,400,318
Cigarette tax	0	900,000,000	970,239,938
CHI premiums	0	555,000,000	555,000,000
Total	0	2,031,294,423	2,377,549,753
