# Supplementary material for: Croatian 2008-2010 health insurance reform: hard choices toward financial sustainability and efficiency
Source: Croat Med J. 2012 Feb;53(1):66–76. doi: 10.3325/cmj.2012.53.66 (PMC3284176; doi:10.3325/cmj.2012.53.66)
Supplement: Supplementary Tables 9 and 10 [file CroatMedJ_53_s009.pdf]

Supplementary Table 9: Standard, minimum and maximum number of patients per team in primary health care. Source of information: reference (33)

Activity	Minimum number of patients per team	Standard number of patients per team	Maximum number of patients per team	Average number of patients per team on April 30, 2011
General Practitioner	1,275	1,700	2,125	1,664
Paediatrics	715	950	1,190	923
Obstetrics and Gynaecology	4,500	6,000	7,500	5,687
Dental medicine	1,650	2,200	2,750	1,957

Supplementary Table 10: Primary care teams in medical centres and on concessions

	Number of teams according to the national network	Number of teams under contract with HZZO	Number of teams missing	Percentage of teams working as salaried professionals in medical centres	Percentage of teams private concessionaires on April 30, 2011
General Practitioner	2,462	2,316	-146	17.3%	82.7%
Paediatrics	323	261	-62	17.7%	82.3%
Obstetrics and Gynaecology	326	267	-59	31.2%	68.8%
Dental medicine	2,209	1,919	-290	13.4%	86.6%
