# Supplementary material for: Croatian 2008-2010 health insurance reform: hard choices toward financial sustainability and efficiency
Source: Croat Med J. 2012 Feb;53(1):66–76. doi: 10.3325/cmj.2012.53.66 (PMC3284176; doi:10.3325/cmj.2012.53.66)
Supplement: Supplementary Table 11 [file CroatMedJ_53_s010.pdf]

Supplementary Table 11: Average duration of the treatment in acute hospitals

Year	Method of payment	Clinical and University hospitals		General hospitals	
		Average duration	Change*	Average duration	Change*
2007	FFS, PPTP	8.6		7.5	
2008	FFS, PPTP	8.5	-1%	7.4	-1%
2009	DTS	7.9	-8%	7.0	-6%
2010	DTS	8.0	1%	7.1	1%
2011 (Jan - Jun)	DTS	7.7	-4%	7.0	-1%

*Comparison with the previous year
