# Supplementary figures and images for: Croatian 2008-2010 health insurance reform: hard choices toward financial sustainability and efficiency
Source: Croat Med J. 2012 Feb;53(1):66–76. doi: 10.3325/cmj.2012.53.66 (PMC3284176; doi:10.3325/cmj.2012.53.66)

Supplementary Figure 1 – Schematic representation of health insurance in Croatia

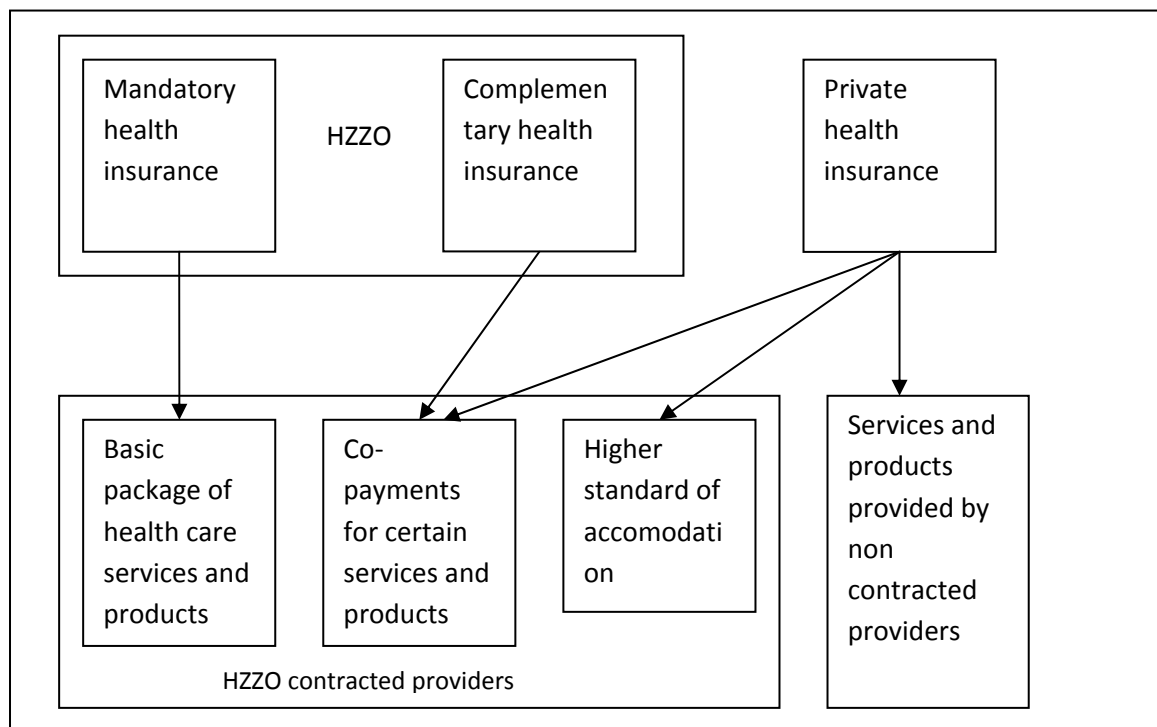

Supplement: Supplementary Figure 1 [file CroatMedJ_53_s011.pdf]
